# GSA ACTIVITIES TO SUPPORT CAREER DEVELOPMENT: RELEVANCE TO DIRECTORS OF AGING CENTERS

**DOI:** 10.1093/geroni/igac059.1343

**Published:** 2022-12-20

**Authors:** Patricia D'Antonio

**Affiliations:** The Gerontological Society of America, Washington, District of Columbia, United States

## Abstract

GSA continues to support our members through career development activities. These activities are designed to empower members to develop the necessary skills to advance their careers as leaders in the field of aging. Through activities like the GSA NIA R13 Diversity, Mentoring, and Career Development Technical Assistance Workshop, we strive to support, promote, and advance the training of diverse students in aging. This presentation will discuss how the experiences with this R13 project and other career development efforts inform additional activities of the Society through a well-framed aging lens. We also discuss the process of soliciting funding to support leadership training and the role that GSA can play in the application process.

